# Transferability of Intra- and Inter-Sectoral Coordination Between Training Tasks and Official Matches in Semi-Professional Football Players

**DOI:** 10.3390/jfmk11030364

**Published:** 2026-09-09

**Authors:** Eduardo Maio, José E. Teixeira, Luís Branquinho, António J. Silva, Pedro Forte, Ricardo Ferraz

**Affiliations:** 1 Department of Sports Sciences, University of Beira Interior, 6201-001 Covilhã, Portugal; ricardo.ferraz@ubi.pt; 2 Research Center in Sports, Health and Human Development, 6201-001 Covilhã, Portugal; luisbranquinho@ipportalegre.pt (L.B.); ajsilva@utad.pt (A.J.S.); 3 Biosciences Higher School of Elvas, Polytechnic University of Portalegre, 7300-110 Portalegre, Portugal; 4 Department of Sports Sciences, Polytechnic University of Cávado and Ave, 4750-810 Guimarães, Portugal; 5 SPRINT—Sport Physical Activity and Health Research & Inovation Center, 6300-559 Guarda, Portugal; 6 Research Center for Active Living and Wellbeing (Livewell), Universidade Politécnica de Bragança, 5300-253 Bragança, Portugal; pedromiguel.forte@iscedouro.pt; 7 Life Quality Research Center (CIEQV), 2001-964 Santarém, Portugal; 8 Department of Sports, Exercise and Health Sciences, University of Trás-os-Montes e Alto Douro, 5001-801 Vila Real, Portugal; 9 Department of Sports Sciences, Universidade Politécnica de Bragança, 5300-253 Bragança, Portugal; 10 Department of Sports, Douro Higher Institute of Educational Sciences, 4560-708 Penafiel, Portugal

**Keywords:** positional data, tactical behavior, training-to-match, training load, soccer

## Abstract

**Background**: This study examined training-to-match transferability of positional coordination in semi-professional football players, comparing responses between defensive and midfield/offensive positional sectors. **Methods**: Twenty-nine players were monitored during the 2023–2024 season. Positional data and external load were collected using global positioning system (GPS) technology. Technical–tactical key performance indicators (KPI) were obtained from SportsBase. Internal training load was assessed using the rating of perceived exertion (RPE), and wellness status was evaluated using the Hooper Index (HI). **Results**: Official matches were associated with greater total distance, average and maximum speed, speed events, acceleration and deceleration events, and mechanical power outputs (*p* < 0.001), with the largest effects for maximum speed (*d* = 1.25), deceleration events (*d* = 1.14), speed events (*d* = 1.13), and relative mechanical power (*d* = 1.05). Positional responses showed distinct training-to-match patterns. In the defensive sector, the official matches showed higher spread rate (SR; *p* = 0.031, *d* = −1.83) and contraction time (CT; *p* = 0.007, *d* = −2.20). In the midfield/offensive sector, matches showed greater effective playing space, width, length, longitudinal synchronization, distance to sector centroid, and stretch index (*p* = 0.005–0.017; *d* = 1.73–2.61), whereas dyadic-distance variability was greater during training (*p* < 0.001, *d* = 4.46). Linear mixed-effects models confirmed greater locomotor and high-intensity demands during matches, while positional differences were more selective. No statistically robust tactical-sector differences were observed for KPIs, internal load, or wellness after adjustment for multiple comparisons. **Conclusions**: Training-to-match transferability was positional sector-dependent, with greater spatial expansion and longitudinal coordination in offensive players and higher overall locomotor demands during matches.

## 1. Introduction

Football performance emerges from the continuous interaction of players within a complex adaptive system, where collective behavior results from the dynamic coupling between teammates, opponents, and contextual constraints [1,2]. Within this framework, team coordination is considered a fundamental mechanism underpinning tactical organization, occurring both within tactical sectors (intra-sectoral coordination) and between sectors (inter-sectoral coordination) to maintain functional team structures during offensive and defensive phases of play [3,4]. The collective behavior coordination represents the continuous evolution of intra- and inter-sectoral relationships, through which players and tactical lines dynamically adjust their relative positioning, spacing, and synchronization to preserve functional team organization across offensive and defensive phases of play [5].

The increasing availability of tracking technologies has substantially advanced the study of football performance. Positional data derived from global positioning systems (GPS), local positioning systems (LPS), and optical tracking systems have enabled the quantification of collective tactical behaviors through variables such as team centroid, interpersonal distances, synchronization, playing space, surface area, and team dispersion [2,5,6]. Recent systematic reviews have highlighted that these positional variables provide valuable insights into team organization and collective adaptations to tactical constraints, offering a more comprehensive understanding of performance than traditional physical metrics alone [5]. Simultaneously, physical performance monitoring has become a key research topic of elite football practice, with metrics such as total distance, high-speed running, sprinting distance, accelerations, and decelerations widely used to quantify external load during training and competition [6]. Collectively, this multidimensional approach integrates positional behavior and coordination, external load, technical–tactical performance, internal load, wellness status, and contextual factors, allowing for a more comprehensive interpretation of how training demands relate to the complex requirements of official competition.

The integration of GPS-derived external load metrics, technical–tactical performance indicators (KPI), perceived exertion responses (RPE), and wellness measures has become increasingly important for understanding the multidimensional demands imposed on different tactical sectors in football [7,8]. Consequently, recent research has advocated for an integrated approach combining positional and physical data to better capture the multidimensional nature of football performance [9,10]. From an ecological dynamics perspective, training tasks should reproduce the informational and tactical constraints encountered during competition to facilitate behavioral transfer [11,12]. Small-sided and conditioned games have, therefore, been extensively used to manipulate tactical and physical demands while maintaining representative learning environments [13,14]. The integration of GPS-derived external load metrics, technical–tactical performance indicators (KPI), perceived exertion responses (RPE), and wellness measures has become increasingly important for understanding the multidimensional demands imposed on different tactical sectors in football [7,8]. Consequently, recent research has advocated for an integrated approach combining positional and physical data to better capture the multidimensional nature of football performance [9,10]. From an ecological dynamic’s perspective, training tasks should reproduce the informational and tactical constraints encountered during competition to facilitate behavioral transfer [11,12]. Small-sided and conditioned games have, therefore, been extensively used to manipulate tactical and physical demands while maintaining representative learning environments [13,14].

Nevertheless, previous research revealed differences in player football movement between training tasks and game behaviors, positional interactions, and training load responses [15,16]. In fact, within the present evidence base, only Nieto et al. [17] contrasted reduced-length training formats with the full competitive pitch format. Moreover, recent evidence suggests that manipulating task constraints can substantially alter both physical workload and tactical organization, highlighting the importance of understanding the representativeness of training environments [16,18]. Despite the substantial body of literature examining tactical behavior and training load independently, the integration of positional and physical data across training and competition remains limited. The recent systematic review by Rico-González et al. [19] emphasized the need for studies investigating how tactical behaviors derived from positional data interact with physical performance, while the review by Teixeira et al. [20] highlighted that training load research continues to rely predominantly on isolated physical metrics, with insufficient attention given to collective tactical adaptations and positional interactions. Consequently, it remains unclear whether the positional behaviors and coordination patterns developed during training are effectively reproduced during official competition and whether these relationships differ according to tactical sectors.

Direct assessment of collective coordination and behavior requires positional information capable of distinguishing within-sector spatial organization from between-sector coupling, while contextualizing these positional patterns within the broader physical and tactical demands [20]. Intra-sectoral coordination reflects the degree to which players belonging to the same sector regulate their relative positioning, spatial dispersion, and movement synchronization [16,18]. Inter-sectoral coordination concerns the maintenance and adjustment of functional spatial relationships between direct sector-to-sector measures, such as the relative distance and synchronization between sector centroids [17,18]. Despite the increasing use of positional tracking technologies in football, evidence examining the extent to which these complementary levels of collective coordination are reproduced from training tasks to official competition remains limited. Previous studies have used positional data derived from the raw data of tracking devices to quantify tactical coordination and behavior [19,20]. These positional data should be complemented by a comprehensive analysis of training load, technical–tactical performance, and wellness indicators to provide a multidimensional interpretation of training-to-match transferability [18,19]. Addressing this research gap may provide important insights to optimize task design and improve the transfer of training adaptations to competition.

Therefore, the primary aim of this study was to examine the training-to-match transferability of the intra- and inter-sectoral coordination in semi-professional football players within a multidimensional monitoring framework integrating positional data, external and internal training load, technical–tactical KPIs, and wellness status. Specifically, positional coordination patterns observed during training tasks were compared with those recorded during official matches to determine whether training-to-match responses differed according to tactical sector.

## 2. Materials and Methods

### 2.1. Study Design

This study followed an observational, longitudinal design conducted throughout the 2023–2024 competitive season. The multidimensional approach adopted in this study integrated positional behavior and collective coordination, external training load, technical–tactical KPI, internal training load, wellness status, and match-related contextual factors, providing a comprehensive characterization of the demands associated with training and official competition [5,7].

### 2.2. Participants

A total of 29 professional football players from a Portuguese Liga 3 team were monitored longitudinally throughout the competitive season and were grouped according to their playing position, and a two-tactical sector classification was retained. The defensive sector included central backs (CB) and full backs (FB) (*n* = 10). he midfield/attacking sector comprised central midfielders (CM) (*n* = 9), wide midfielders (WM) (*n* = 5), and forwards (FW) (*n* = 5). Overall, the sample consisted of 5 CB (17.2%), 5 FB (17.2%), 9 CM (31.0%), 5 WM (17.2%), and 5 FW (17.2%). This classification ensured representation of all tactical sectors, allowing for comparisons between players performing distinct tactical roles throughout the training and monitoring observation period.

The inclusion criteria were (i) completing the entire match under analysis; (ii) wearing a valid GPS device throughout the entire match; and (iii) being free from injury during the data collection period. Goalkeepers and players with incomplete data were excluded due to their distinct physical and tactical demands. In addition, players with incomplete or invalid GPS data, those who did not complete the full match (e.g., substituted in or out), and players who sustained an injury during the observation period were excluded from the analysis. The study was conducted in accordance with the Declaration of Helsinki and approved by the Ethics Committee at the University of Beira Interior (CE-UBI-Pj-2024-067-ID2595). All participants provided informed consent before participation.

### 2.3. Data Collection

Data were collected longitudinally across the 2023–2024 competitive season, including 10 competitive microcycles, 50 training sessions (five sessions per microcycle), and 10 official matches. A total of 847 observations were collected during the study period. Of these, 705 observations were obtained during official matches, and 142 training observations were obtained per week, reporting 710 training observations per week in total. Each observation corresponded to an individual player exposure recorded through the club’s athlete monitoring system. Before the commencement of the official data collection, the researchers completed standardized training sessions on the placement, activation, and monitoring of the training load and tactical performance, ensuring methodological consistency throughout the entire data collection process for external training load, positional tactical behavior, internal training load, wellness status, match-related KPI and contextual factors. Differences in the number of valid observations across variables and monitoring domains were mainly attributable to incomplete data collection at the individual-player level. In training, some exposures were incomplete because players did not complete the full session due to coaching decisions, temporary absence, injury, or other participation-related constraints. In addition, RPE and wellness datasets contained occasional missing observations due to non-response to the corresponding questionnaires. Consequently, the number of valid observations differed between the variables and the conditions. This poor-quality data was taken from training and match load monitoring. Only observations meeting the predefined completeness and validity criteria for each specific outcome were included in the analyses, and no missing values were imputed.

#### 2.3.1. External Training Load

The external training load was collected using GPS technology during regular training sessions and official matches throughout the competitive season [9,21]. The data were obtained from the GPS (Global Positioning System), specifically the 18.18 Hz GPEXE^®^ GPS devices (Exelio SRL, Udine, Italy), and were recorded and processed using the GPEXE online application (version 8.2.41, Exelio SRL, Udine, Italy). This sampling frequency reflected the hardware acquisition rate and was not obtained through software interpolation or post-processing up-sampling. The variables analysed and included were duration (DUR), total playing time (TPT), total distance (TD), average speed (AvgS), maximum speed (MaxS), high-speed running distance (HSRD; >19.8 km·h^−1^), sprint distance (SPD; >25.2 km·h^−1^), speed events (SE), acceleration events (AccEv; >3.0 m·s^−2^), deceleration events (DecEv; <−3.0 m·s^−2^), maximum acceleration (AccMax), maximum deceleration (DecMax), mechanical power estimate (MPE), and relative mechanical power (rMPE). All GPS data were extracted and processed according to the manufacturer’s recommendations and following current guidelines for external load monitoring in football. The validity and reliability of the tracking system for football-specific locomotor and positional monitoring were considered according to previous validation studies [6,22].

#### 2.3.2. Positional Data and Collective Coordination

Player positional coordinates were obtained from the tracking system and processed to quantify intra- and inter-sectoral spatial organization and collective coordination. Players were grouped according to their tactical sector (defensive, midfield, and attacking), and positional metrics were calculated from the two-dimensional longitudinal and lateral coordinates recorded throughout each eligible training and match exposure [20]. Player positional coordinates were obtained from the same tracking system and processed to quantify intra- and inter-sectoral spatial organization during training tasks and official matches. Positional variables were computed continuously from the player coordinate time series and summarized for each eligible exposure. For training, positional metrics were aggregated at the player–week/microcycle level according to the same longitudinal procedure applied to the external-load variables [18,19]. Match positional metrics were retained at the player–match level. All positional variables were processed using MATLAB routines, version 26.1 (MathWorks, Inc., Natick, MA, USA) [23].

Particularly, distance between dyads (m) was calculated as the Euclidean distance between all possible pairs of players belonging to the same tactical sector at each frame. To quantify the relative variability of these interpersonal distances, the coefficient of variation of distance between dyads (CV, %) was calculated for each dyad as 100 × standard deviation divided by mean distance, with the sector-level value obtained as the mean of all valid dyads [24,25]. Effective playing space (EPS, m^2^) was calculated at each frame as the area of the minimum convex hull delimited by the players belonging to the corresponding tactical sector. Width (m) represented the difference between the maximum and minimum lateral coordinates within the sector, whereas Length (m) represented the difference between the maximum and minimum longitudinal coordinates.

Inter-player movement synchronization was quantified separately in the longitudinal and lateral directions [23]. Longitudinal Synchronization (%) was calculated from the relative phase between all possible player dyads using the Hilbert transform applied to the longitudinal coordinate time series. Dyads were in near-in-phase synchronization when the relative phase values were between −30° and +30°, and the outcome was expressed as the percentage of time spent within this range. Lateral synchronization (%) was calculated using the same procedure applied to the lateral coordinate time series. The distance to sector centroid (m) was calculated as the Euclidean distance between each player and the dynamically computed centroid of the corresponding tactical sector at each frame [3,26]. The stretch index (SI) (m) was subsequently calculated as the mean distance of all sector players from the sector centroid at each frame, providing an estimate of sectoral spatial dispersion [27,28]. The temporal change in sectoral dispersion was quantified using the Spread Rate (SR) (m·s^−1^), calculated as the temporal derivative of the Stretch Index, with the mean absolute value used to characterize the magnitude of expansion and contraction dynamics. Dispersion time (DI) (%) was defined as the percentage of frames in which the spread rate (SR) was greater than zero, indicating sectoral expansion, whereas the contraction time (CI) (%) represented the percentage of frames in which the spread rate was lower than zero, indicating sectoral contraction. The positional variables were used to characterize the spatial structure, variability, synchronization, and expansion–contraction dynamics of the tactical sectors [27,29]. They were analyzed alongside external load, technical–tactical performance, internal load, wellness status, and contextual factors within the multidimensional framework of the study.

Player selection for the positional analysis was based on the availability and completeness of the data recorded for each match. Within the defensive sector, the four players with the highest number of valid positional samples or cells were selected, whereas the offensive sector, comprising midfielders and forwards, included the six players with the highest number of valid records. Selection was performed exclusively among the files corresponding to the match and date under analysis; files associated with different dates or matches were excluded and documented as methodological exclusions. To ensure procedural traceability, all eligible players were ranked according to the number of valid samples and recorded in the Player_Coverage sheet, including their ranking position and selection status (Selected = Yes/No). The variables were analyzed according to the team sector to subsequently assess the differences between intra- and inter-coordination.

#### 2.3.3. Key Performance Indicators (KPI)

The match-related KPI data were obtained from the cloud-based football analytics platform SportsBase (SportsBase Hungary Kft., Budapest, Hungary) and were accessed and processed using the SportsBase web application (SportsBase. Available online: https://sportsbase.world/ (accessed on 25 August 2026)). The platform provides standardized match event data and advanced technical and tactical performance indicators from official football [2,30]. These KPI indicators were used to characterize players’ technical and tactical contributions during official competitions, specifically goals (G), assists (A), shots (SH), shots on target (SoT), chances created (CC), total passes (TP), successful passes (SP), pass accuracy (PA), key passes (KP), progressive passes (PP), crosses (CR), successful crosses (SCR), offensive duels (OD), defensive duels (DD), interceptions (INT), ball recoveries (BR), ball losses (BL), fouls committed (FC), and fouls suffered (FS). These variables were used to characterize players’ offensive and defensive technical–tactical contributions during official matches.

#### 2.3.4. Wellness Status

Players’ wellness statuses were assessed daily using the Hooper Index (HI) questionnaire [31]. The questionnaire was completed individually before the first daily training activity under the supervision of the coaching staff, ensuring standardized data collection procedures. comprising sleep quality (SLP), stress (STR), fatigue (FAT), delayed onset muscle soreness (DOMS), hydration status (HYD), and the overall HI score [31]. Each dimension was rated using a 7-point Likert scale, where lower scores indicated a more favorable wellness status (e.g., very good sleep quality, very low stress, very low fatigue, or absence of muscle soreness), whereas higher scores reflected poorer perceived recovery and greater physiological or psychological strain. Individual scores from the four dimensions were summed to obtain the overall HI score, with higher values indicating poorer wellness status and a greater likelihood of accumulated fatigue.

#### 2.3.5. Internal Training Load

Internal load was monitored using the Rating of Perceived Exertion (RPE) method. Session Rating of Perceived Exertion (sRPE) was subsequently calculated by multiplying the RPE score by session duration, providing an estimate of the overall internal training load [32,33]. The Borg CR-10 scale was used to collect players’ perceived exertion responses following training sessions and matches. Previous research has demonstrated that sRPE is a valid and reliable measure for monitoring internal load in football players and is strongly associated with physiological and external load responses [34,35].

#### 2.3.6. Training Microcycle and Match-Related Contextual Factors

Training microcycles were organized using the match-day-minus approach (MD−5 to MD-1 [36,37]. Training contents were progressively adjusted according to proximity to competition: on MD-3, training emphasized offensive circulation, crossing, possession, and collective tactical organization; on MD-2, the focus shifted to finishing, defensive organization, offensive transitions, and speed-oriented activities; and on MD-1, training involved lower-volume, match-specific work, including offensive set pieces and reaction-speed tasks. MD corresponded to the official game [7,38,39].

### 2.4. Variables

**Figure 1:**
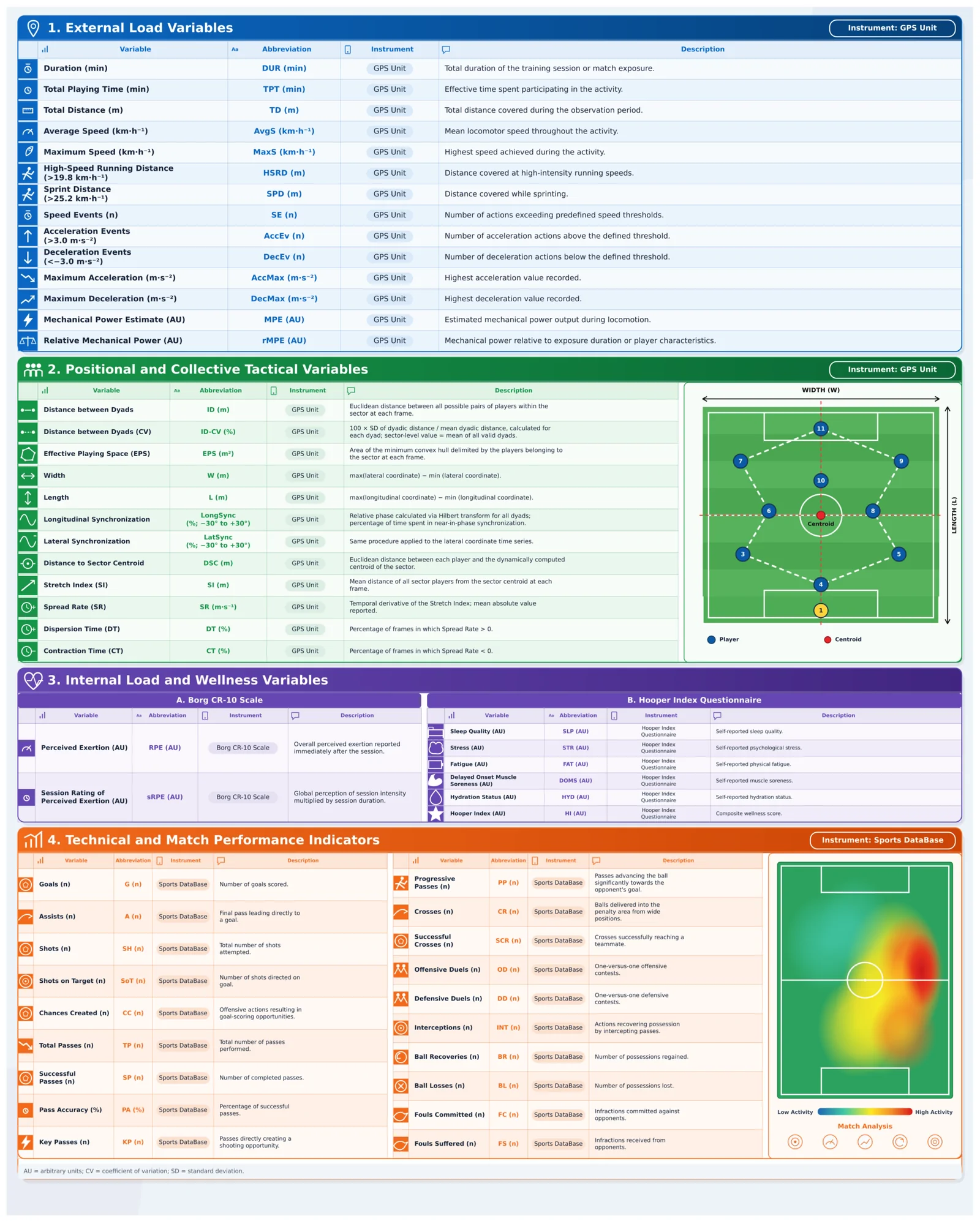
Multidimensional monitoring framework integrating external load, positional metrics, internal load, technical–tactical KPIs, and wellness indicators.

Figure 1 illustrates the multidimensional monitoring framework adopted in the study, integrating external training load, positional and tactical metrics, internal training load, technical–tactical KPIs, and wellness indicators to provide a comprehensive characterization of training-to-match demands in semi-professional football players.

The study variables were organized into two analytical levels. Tactical positional variables directly quantified intra- and inter-sectoral coordination [18,19]. External-load, technical–tactical, internal-load, and wellness indicators were used to contextualize the multidimensional demands associated with training and official competition. The variables were organized into five complementary dimensions [5,40]: (i) positional behavior and collective coordination (distance between dyads (DBD), distance between dyads—coefficient of variation (DBD-CV), effective playing space (EPS), width (W), length (L), longitudinal synchronization (LongSyn), lateral synchronization (LatSyn), distance to sector centroid (DSC), stretch index (SI), spread rate (SR), dispersion time (DI), and contraction time (CI)); (ii) external load (DUR, TPT, TD, AvgS, MaxS, HSRD, SPD, SE, AccEv, DecEv, AccMax, DecMax, MPE, and rMPE); (iii) technical–tactical performance (G, A, SH, SoT, CC, TP, SP, PA, KP, PP, CR, SCR, OD, DD, INT, BR, BL, FC, and FS); (iv) internal load (RPE and sRPE); and (v) wellness status (SLP, STR, FAT, DOMS, HYD, and HI). Positional variables were considered the primary outcomes for directly quantifying intra- and inter-sectoral coordination, whereas the remaining dimensions were analyzed as complementary indicators of the multidimensional demands associated with training and official competition.

### 2.5. Statistical Analysis

Statistical analyses were performed using IBM SPSS Statistics (Version 25; IBM Corp., Armonk, NY, USA). Descriptive statistics were calculated and are presented as the mean ± standard deviation (SD). Data normality was assessed using the Shapiro–Wilk test and homogeneity of variances using Levene’s test. To compare the external load variables between training sessions and official matches, independent-sample *t*-tests were performed for all GPS-derived variables. Additional independent *t*-tests were conducted to compare technical–tactical KPI, RPE [41], and wellness variables between defensive and offensive sectors. Effect sizes were calculated using Cohen’s d and interpreted as trivial (<0.20), small (0.20–0.59), moderate (0.60–1.19), large (1.20–1.99), and very large (≥2.00) [42]. To account for the longitudinal repeated-measures structure, linear mixed-effects models (LMM) were additionally fitted for eligible positional data, external and internal training load, and technical–tactical KPI and wellness indicators. Players were included as a random intercept, while context (training vs. official match), tactical sector (defensive, midfield, attacking), and their interaction were entered as fixed effects. LMM models were estimated using restricted maximum likelihood, with training and the defensive sector as reference categories. Adjusted match–training contrasts were reported with 95% confidence intervals, and intraclass correlation coefficients (ICC) were calculated to quantify between-player variability. Holm adjustment was applied across LMM outcomes to control for multiple comparisons. The level of statistical significance was set at *p* < 0.05.

## 3. Results

### 3.1. Comparison of GPS-Based External Training Load and Tactical Positional Variables Between Defensive and Offensive Sectors

The comparison between training sessions and official matches revealed substantial differences in external load demands experienced by semi-professional football players (Table 1). Overall, official matches elicited significantly greater locomotor and mechanical demands than training sessions. Match play was associated with greater duration (DUR; *p* < 0.001, *d* = −0.38), total distance covered (TD; *p* < 0.001, *d* = −0.51), average speed (AvgS; *p* < 0.001, *d* = −0.51), and maximum speed attained (MaxS; *p* < 0.001, *d* = −1.25). The largest effect size was found for maximum speed, indicating that official matches expose players to considerably greater peak locomotor intensities than training sessions. These findings suggest that, although training activities may reproduce certain tactical and technical demands, they may not fully replicate the maximal speed requirements encountered during competition.

Table 2 showed the comparison between defensive and offensive sectors for positional data variables. The training-to-match response differed markedly between the defensive and midfield/offensive sectors (Table 2). In the defensive sector, significant differences between training and match conditions were limited to SR index, which was higher during matches (0.477 ± 0.058 vs. 0.368 ± 0.063 m·s^−1^; *p* = 0.031, *d* = −1.83), and CT, which was also higher during matches (50.31 ± 1.43% vs. 47.48 ± 1.02%; *p* = 0.007, *d* = −2.20). In contrast, the midfield/offensive sector showed a broader training-to-match differentiation, with matches displaying greater EPS, width, length, longitudinal synchronization, distance to sector centroid, and SI (*p* = 0.005–0.017; *d* = 1.73–2.61), whereas the coefficient of variation of distance between dyads was substantially greater during training (*p* < 0.001, *d* = 4.46). No significant training-to-match differences were observed for lateral synchronization in either sector, while the distance between dyads and dispersion time also remained unchanged. Overall, these findings indicate that the midfield/offensive sector exhibited greater spatial expansion and longitudinal coordination during competition relative to training, whereas the defensive sector showed comparatively greater stability across conditions, with differences primarily related to the dynamics of dispersion and contraction.

### 3.2. Comparison of Technical–Tactical Performance Indicators Between Defensive and Offensive Sectors

The technical–tactical performance indicators differed substantially between tactical sectors (Table 3). Offensive-sector players presented significantly greater values for SH, SoT, KP, PP, OD, and FS, whereas defensive-sector players recorded significantly greater values for TP, SP, DD, INT, and BR. The largest effect sizes were observed for interceptions (*d* = 1.19), progressive passes (*d* = 0.99), total passes (*d* = 0.92), offensive duels (*d* = −0.91), and successful passes (*d* = 0.88), indicating substantial tactical specialization according to playing role.

### 3.3. Comparison of Perceived Exertion and Wellness Indicators Between Defensive and Offensive Sectors

The comparison of self-reported monitoring variables revealed small but significant differences between sectors (Table 4). Defensive-sector players reported slightly higher RPE and hydration scores than offensive-sector players. No significant differences were observed for sleep quality, stress, DOMS, or the overall HI score. Effect sizes were generally trivial-to-small, suggesting limited practical differences in subjective monitoring variables between tactical sectors.

### 3.4. Linear Mixed-Effects Analysis of Training-to-Match

The LMM accounting for repeated observations within players confirmed systematic differences between training and official match play across the GPS-derived outcomes analyzed. After adjustment for tactical sector and multiple comparisons, official matches were associated with 1623.15 m greater total distance (95% CI: 921.22 to 2325.08; Holm-adjusted *p* < 0.001), 1.03 km·h^−1^ greater average speed (95% CI: 0.82 to 1.23; adjusted *p* < 0.001), and 2.90 km·h^−1^ greater maximum speed (95% CI: 2.39 to 3.40; adjusted *p* < 0.001) compared with training. Official matches were also associated with higher frequencies of high-intensity movement events, including 6.11 additional speed events (95% CI: 4.43 to 7.80; adjusted *p* < 0.001), 8.62 additional acceleration events (95% CI: 4.74 to 12.50; adjusted *p* < 0.001), and 18.73 additional deceleration events (95% CI: 13.97 to 23.48; adjusted *p* < 0.001). Maximum acceleration was also higher during official matches, although the magnitude of this difference was smaller (β = 0.19 m·s^−2^, 95% CI: 0.06 to 0.32; adjusted *p* = 0.003).

The positional LMMs revealed a more sector-specific training-to-match pattern. Within the defensive sector, official matches were associated with greater width (β = 9.98 m, 95% CI: 5.23 to 14.73; Holm-adjusted *p* < 0.001) and a greater spread rate (β = 0.133 m·s^−1^, 95% CI: 0.070 to 0.197; adjusted *p* < 0.001). No other positional outcome showed a statistically significant training-to-match contrast in the defensive sector after adjustment for multiple comparisons (all adjusted *p* ≥ 0.05).

In the midfield/offensive sector, official matches were characterized by substantially lower variability in distance between dyads (DBD-CV: β = −18.08 percentage points, 95% CI: −25.22 to −10.94; adjusted *p* < 0.001), together with greater effective playing space (EPS: β = 191.44 m^2^, 95% CI: 72.63 to 310.24; adjusted *p* = 0.013), width (β = 11.96 m, 95% CI: 7.21 to 16.71; adjusted *p* < 0.001), and length (β = 9.59 m, 95% CI: 2.79 to 16.38; adjusted *p* = 0.040). Official matches were also associated with a greater distance to the sector centroid (β = 3.21 m, 95% CI: 1.32 to 5.09; adjusted *p* = 0.008) and, consequently, a greater stretch index (β = 3.21 m, 95% CI: 1.32 to 5.09; adjusted *p* = 0.008). No adjusted training-to-match differences were identified for longitudinal synchronization, lateral synchronization, spread rate, dispersion time, contraction time, or mean distance between dyads within this sector. The mixed-effects analyses showed no statistically robust tactical-sector differences in KPI, internal load or wellness after adjustment for multiple comparisons.

The context–tactical sector interaction did not remain statistically significant for any outcome after Holm correction (all adjusted *p* ≥ 0.075), indicating that the overall training-to-match differences were broadly consistent across defensive, midfield, and attacking sectors. Player-level ICC values ranged from 0.10 to 0.39, demonstrating meaningful clustering of repeated observations within players and supporting the use of a mixed-effects modelling approach.

## 4. Discussion

This study investigated the training-to-match transferability of positional coordination in semi-professional football players, with particular emphasis on differences between defensive and midfield/offensive positional sectors. The present study showed that training-to-match transferability was characterized by marked differences in both physical and positional demands. Official matches imposed substantially greater locomotor and mechanical demands than training, particularly for maximum speed, speed events, deceleration events, and relative mechanical power. Positional responses, however, showed a more selective pattern. The defensive sector showed relatively stable positional behavior across contexts, with significant differences limited to spread rate and contraction time. Conversely, the midfield/offensive sector showed greater spatial expansion and longitudinal synchronization during matches, whereas training elicited greater variability in dyadic distances. LMM models confirmed these external-load differences after accounting for repeated observations within players, with no significant context–tactical sector interactions after correction. Overall, the training and match contexts differed across several positional and physical outcomes, with the most pronounced positional differences in spatial expansion observed within the midfield/offensive analytical group.

The findings indicate that official matches impose substantially greater physical and mechanical demands than training sessions across most GPS-derived variables. The moderate-to-large effect sizes observed for maximum speed, speed events, deceleration events, and relative mechanical power further suggest that training tasks were associated with lower exposure to several of the most demanding locomotor actions observed during competition. Therefore, practitioners may consider whether the weekly training program provides sufficient exposure to the intensity and frequency of high-speed and high-power actions observed during match play [43,44]. The greater total distance covered during official matches is consistent with previous research conducted in professional and semi-professional football, which has consistently demonstrated that competition is generally associated with higher locomotor demands than training sessions. Match play requires players to continuously adapt to the unpredictable nature of opposition behavior, tactical transitions, and game-specific contexts, leading to greater overall movement demands. In contrast, training sessions are frequently constrained by pitch dimensions, task objectives, coach interventions, and work-to-rest ratios, which may reduce the total volume of locomotor activity [45,46].

One of the most notable findings was the substantially greater maximum speed attained during matches, with a large effect size. This result aligns with previous studies reporting that sprinting actions are among the most difficult physical demands to reproduce during training [46,47]. Although small-sided games and tactical drills are effective for developing technical and tactical behaviors, they often limit opportunities for players to perform maximal sprinting actions due to reduced playing areas and increased player density. Consequently, players may receive lower exposure to peak velocity demands during routine training, which may have implications for physical preparation. The present findings indicate that the midfield/offensive positional group displayed a substantially more expansive and longitudinally coordinated spatial organization during matches than during training, as reflected by the higher EPS, W, L, DSC, SI, and LongSync values observed in competition. This pattern is consistent with previous evidence showing that the spatial and task constraints imposed during training strongly shape collective tactical behavior [14,16,17]. Specifically, increasing the relative pitch area has been shown to promote greater spatial occupation and team dispersion, thereby increasing tactical demands and improving the representativeness of training relative to match play [16]. Likewise, variations in side-game formats can substantially modify players’ external and spatial demands, indicating that constrained training tasks may not fully reproduce the demands imposed by competition [13]. The markedly higher DBD-CV observed during training may, therefore, reflect greater fluctuations in interpersonal spacing and more frequent reorganization of player-to-player relationships within comparatively smaller spatial configurations. Training variability may be influenced by task constraints, including playing areas, coaches’ instructions, and game directionality, which have been shown to modify players’ positioning and collective tactical behavior [14,15,17]. Coaches’ instructions can promote either more compact or more expansive team behaviors [15], while manipulating directionality may alter space exploration and interpersonal positioning [17]. Future predictive modeling approaches should, therefore, consider task constraints and contextual variables alongside positional and load-related outcomes. Similarly, official matches were characterized by a significantly greater number of speed events and deceleration actions. The large effect sizes observed for both variables indicate that competition exposes players to more frequent explosive actions and rapid changes in movement intensity. These findings corroborate previous research demonstrating that accelerative and decelerative load represent critical components of football performance and contribute substantially to neuromuscular fatigue [45,46]. Decelerations have been associated with elevated eccentric muscle loading and may constitute a major determinant of post-match fatigue and recovery requirements. Mechanical load variables also displayed marked contextual differences. Both MPE and rMPE were significantly greater during official matches, suggesting that competitive environments impose higher mechanical and metabolic demands than training sessions. Previous studies have shown that mechanical power-related metrics are strongly associated with high-intensity running, repeated accelerations, and changes in direction, all of which occur more frequently during competition [5,26]. Therefore, the higher values observed during matches likely reflect the combined influence of greater movement intensity and a more dynamic tactical environment. However, the extremely large variability recorded for match observations suggests the presence of substantial heterogeneity within the dataset, potentially resulting from differences in playing time exposure, substitutions, or recording procedures. Consequently, this variable should be interpreted cautiously and may not accurately reflect meaningful contextual differences between training and competition.

The present findings have several practical implications for coaches, performance analysts, sport scientists, and strength and conditioning practitioners, particularly regarding the use of positional data to evaluate training transfer and task representativeness. Direct positional monitoring provides information that extends beyond conventional external load metrics by allowing practitioners to examine the extent to which training tasks reproduce the spatial organization and collective behaviors observed during official competition [13,14,15,16,17]. From a physical perspective, routine football training may not consistently reproduce the highest locomotor, and sprint demands imposed by competitive match play [9,46], reinforcing the importance of considering high-speed exposure when evaluating the representativeness of the weekly training process. The monitoring of acceleration, deceleration, and mechanical power can further complement this assessment by characterizing the mechanical and neuromuscular demands imposed across training and competition [45]. Where routine football practice provides insufficient exposure to high-speed and sprint actions, appropriately planned supplementary speed-oriented interventions may, therefore, represent a complementary training strategy [37,38]. From a positional perspective, the observed training-to-match differences in spatial occupation, interpersonal organization, and expansion–contraction dynamics indicate that external-load correspondence alone may not adequately represent the specificity of the training environment.

Collectively, the present findings reinforce the growing body of evidence indicating that training sessions may successfully reproduce some aspects of football-specific activity but frequently underestimate the most demanding locomotor and mechanical actions experienced during competition. This discrepancy is particularly evident for variables associated with maximal speed, high-intensity movement events, and mechanical power production. As such, coaches and performance practitioners should carefully consider the inclusion of complementary training strategies specifically designed to expose players to match-like sprinting and high-intensity locomotor demands. Overall, the present results suggest that the training displayed some of the interpersonal variability observed during competition but was characterized by lower spatial expansion and longitudinal synchronization for selected positional outcomes. This interpretation is consistent with systematic evidence supporting the use of positional measures to quantify collective tactical behavior [18,19] and highlighting the importance of integrating training- and match-based positional data when assessing the representativeness of football training tasks [19].

### Limitations, Future Research Directions and Practical Applications

Several limitations should be acknowledged when interpreting the findings of this study. First, the data were collected from a single semi-professional football team, which may limit the generalizability of the results to other competitive levels, playing styles, or tactical systems. Similar limitations have been highlighted in previous football monitoring studies investigating training and match demands [22,40]. Second, the observational nature of the study prevents causal inferences regarding the factors responsible for the observed differences between training and match contexts [7]. Third, contextual variables such as match location, opposition quality, scoreline, and tactical formations were not considered and may have influenced the external load responses, as previously reported in football performance research [43]. Finally, some variables presented substantial variability and unequal sample sizes between training and match observations, which may have influenced statistical sensitivity and reduced the precision of the estimated effects [44]. Although collective coordination was directly examined using positional data, the available metrics predominantly characterized spatial organization and coordination within the predefined positional groups. Direct measures of between-sector coupling, such as the relative distance and synchronization between defensive, midfield, and attacking sector centroids, were not included and should be considered in future research. [20]. Consequently, the findings should be interpreted as evidence of training-to-match differences in the positional dimensions examined, rather than as a comprehensive assessment of all dimensions of intra- and inter-sectoral coordination. Future studies should extend the present positional framework by incorporating direct inter-sectoral measures, including distances and synchronization between tactical-sector centroids, to more comprehensively quantify the coordination between defensive, midfield, and attacking lines during official match play. Future investigations should examine contextual differences across multiple teams, competitive levels, and playing philosophies to improve the external validity of the findings [39]. Also, future research should extend the present training-to-match framework by examining positional and physical responses hierarchically according to session type, exercise format, relative playing area, numerical configuration, microcycle day, and other relevant contextual constraints [47]. The integration of physical, physiological, technical and tactical factors may provide a more comprehensive understanding of how training environments reproduce competitive demands [39,45]. Longitudinal studies incorporating machine learning approaches and multivariate performance models may further identify the factors that contribute most strongly to successful transfer between training and competition [15,31]. Finally, future research should explore the interaction between external training load, wellness, perceived exertion, and technical performance indicators to better understand the multidimensional nature of football performance [37]. Although unadjusted comparisons suggested differences in selected RPE and hydration outcomes, these effects were not statistically robust after adjustment for multiple comparisons, indicating limited evidence of systematic sector-related differences in internal load and wellness.

Furthermore, the observed differences between training and competition reinforce the need to move beyond a generic training-versus-match comparison and to consider the specific characteristics of the training tasks used throughout the microcycle. Small-, medium-, and large-sided games, as well as manipulations of relative pitch area, directionality, numerical configuration, and coaches’ instructions, can substantially modify the spatial, tactical, and physical responses of players [13,14,15,16,17]. Consequently, training representativeness should not be interpreted solely according to whether a given variable differs from the match values, but rather according to the extent to which different exercise formats reproduce the spatial expansion, interpersonal variability, synchronization, and contraction–dispersion dynamics observed during competition [14,16,17,18,19].

## 5. Conclusions

Official match play was characterized by greater locomotor and mechanical demands than training, particularly for maximum speed, speed events, decelerations, and relative mechanical power. Positional responses also demonstrated variable-dependent training-to-match differences. Within-group analyses indicated comparatively stable defensive positional behavior across training and match contexts, whereas the midfield/offensive positional group displayed greater spatial expansion and longitudinal synchronization during official matches than during training. Nevertheless, these within-group patterns should not be interpreted as evidence of uniformly sector-specific transferability, as statistical support for differential responses between positional groups was limited. Technical–tactical, perceived-exertion, and wellness indicators showed limited statistically robust differences between tactical sectors after adjustment for multiple comparisons. Collectively, these findings highlight the value of integrating positional and external-load monitoring within a multidimensional framework to evaluate training representativeness and to inform the design of practice tasks that more closely reproduce the spatial, coordinative, and physical demands of official competition.

## Figures and Tables

**Table 1:** Independent samples test comparing GPS-derived variables between training sessions and official matches in semi-professional football players.

Variable	Training (*n*)	Training (M ± SD)	Match (*n*)	Match (M ± SD)	*t*	*p*	Cohen *d*
DUR (min)	630	64.23 ± 22.88	703	75.55 ± 31.00	−4.82	<0.001	−0.38
TPT (min)	630	69.99 ± 16.15	705	344.77 ± 580.08	−1.26	0.209	−0.05
TD (m)	710	5848.93 ± 1705.16	705	8057.15 ± 4675.58	−9.73	<0.001	−0.51
AvgS (km·h^−1^)	710	5.65 ± 1.52	572	6.40 ± 1.44	−5.30	<0.001	−0.51
MaxS (km·h^−1^)	710	26.10 ± 2.11	705	29.31 ± 2.64	−15.76	<0.001	−1.25
SE (*n*)	250	1.57 ± 1.54	705	7.61 ± 5.50	−20.13	<0.001	−1.13
AccEv (*n*)	250	13.43 ± 9.48	705	33.95 ± 79.89	−6.23	<0.001	−0.27
DecEv (*n*)	250	13.40 ± 11.90	705	30.80 ± 15.49	−9.77	<0.001	−1.14
AccMax (m·s^−2^)	710	4.03 ± 0.37	501	5.39 ± 6.46	−4.67	<0.001	−0.24
MPE (AU)	375	85.35 ± 12.30	170	119.27 ± 48.30	−8.55	<0.001	−0.83
rMPE (AU)	710	31.64 ± 16.57	293	265.44 ± 269.86	−14.77	<0.001	−1.05

Note. AccEv = acceleration events; AccMax = maximum acceleration; AU = arbitrary units; AvgS = average speed; DecEv = deceleration events; DUR = duration; MaxS = maximum speed; MPE = mechanical power estimate; rMPE = relative mechanical power; SE = speed events; TD = total distance; TPT = total playing time.

**Table 2:** Comparison between training and match for the midfield/offensive sector in positional data variables from GPS.

Variable	Training (*n*)	Training (M ± SD)	Match (*n*)	Match (M ± SD)	*t*	*p*	Cohen *d*
ID (m)	710	18.69 ± 2.97	705	21.09 ± 1.61	−1.70	0.129	−0.97
ID-CV (%)	710	60.80 ± 4.79	705	44.60 ± 0.99	8.07	<0.001	4.46
EPS (m^2^)	710	249.79 ± 90.71	705	447.10 ± 56.53	−4.13	0.005	−2.61
W (m)	710	20.22 ± 6.48	705	32.53 ± 5.51	−3.40	0.008	−2.03
L (m)	710	17.07 ± 5.47	705	25.59 ± 4.06	−2.96	0.016	−1.74
LongSync (%)	710	62.76 ± 4.60	705	75.08 ± 6.96	−3.39	0.012	−2.13
LatSync (%)	710	38.09 ± 5.75	705	40.51 ± 5.53	−0.71	0.497	−0.43
DSC (m)	710	10.82 ± 1.92	705	13.57 ± 1.05	−3.01	0.017	−1.73
SI (m)	710	10.82 ± 1.92	705	13.57 ± 1.05	−3.01	0.017	−1.73
SR (m·s^−1^)	710	0.403 ± 0.058	705	0.397 ± 0.022	0.24	0.821	0.13
DT (%)	710	49.74 ± 1.16	705	51.11 ± 1.61	−1.59	0.155	−0.99
CT (%)	710	49.17 ± 1.63	705	48.89 ± 1.61	0.29	0.776	0.18

Abbreviations: CT = contraction time; DSC = distance to sector centroid; DT = dispersion time; EPS = effective playing space; ID = distance between dyads; ID-CV = distance between dyads coefficient of variation; L = length; LatSync = lateral synchronization; LongSync = longitudinal synchronization; M = mean; SD = standard deviation; SI = stretch index; SR = spread rate; W = width.

**Table 3:** Comparison of technical–tactical performance indicators between defensive and offensive sectors in semi-professional football players.

Variable	Defensive Sector (Mean ± SD)	Offensive Sector (Mean ± SD)	*t*	*p*	Cohen’s *d*
G (*n*)	1.00 ± 0.00	1.14 ± 0.45	−1.69	0.103	−0.35
A (*n*)	1.00 ± 0.00	1.05 ± 0.22	−1.00	0.329	−0.24
SH (*n*)	1.43 ± 0.63	1.93 ± 1.24	−4.17	<0.001	−0.44
SoT (*n*)	1.11 ± 0.32	1.33 ± 0.64	−2.52	0.014	−0.37
CC (*n*)	1.14 ± 0.35	1.24 ± 0.50	−1.34	0.187	−0.22
TP (*n*)	35.85 ± 19.55	20.38 ± 15.24	9.93	<0.001	0.92
SP (*n*)	27.24 ± 16.00	15.21 ± 12.35	9.45	<0.001	0.88
PA (%)	0.73 ± 0.16	0.72 ± 0.18	0.86	0.388	0.07
KP (*n*)	1.19 ± 0.40	1.49 ± 0.90	−2.79	0.006	−0.37
PP (*n*)	9.77 ± 5.50	5.21 ± 4.07	10.12	<0.001	0.99
CR (*n*)	2.88 ± 1.89	1.89 ± 1.40	3.99	<0.001	0.64
SCR (*n*)	0.68 ± 0.78	0.39 ± 0.67	2.75	0.007	0.41
OD (*n*)	3.18 ± 2.38	7.74 ± 5.76	−13.48	<0.001	−0.91
DD (*n*)	8.87 ± 4.64	5.37 ± 3.97	9.00	<0.001	0.83
INT (*n*)	6.58 ± 3.55	3.15 ± 2.44	11.73	<0.001	1.19
BR (*n*)	5.43 ± 3.25	4.16 ± 2.92	4.57	<0.001	0.42
BL (*n*)	1.31 ± 0.61	1.18 ± 0.44	1.39	0.167	0.23
FC (*n*)	1.54 ± 0.89	1.66 ± 0.88	−1.14	0.255	−0.13
FS (*n*)	1.47 ± 0.82	1.82 ± 1.06	−3.34	0.001	−0.36

Note. A = assists; BL = ball losses; BR = ball recoveries; CC = chances created; CR = crosses; DD = defensive duels; FC = fouls committed; FS = fouls suffered; G = goals; INT = interceptions; KP = key passes; M = mean; OD = offensive duels; PA = pass accuracy; PP = progressive passes; SCR = successful crosses; SD = standard deviation; SH = shots; SoT = shots on target; SP = successful passes; TP = total passes.

**Table 4:** Comparison of perceived exertion and wellness indicators between defensive and offensive sectors in semi-professional football players.

Variable	Defensive Sector (Mean ± SD)	Offensive Sector (Mean ± SD)	*t*	*p*	Cohen’s *d*
RPE (AU)	4.97 ± 1.84	4.71 ± 1.81	2.21	0.028	0.14
SLP (AU)	2.10 ± 0.78	2.07 ± 0.86	0.50	0.615	0.04
STR (AU)	1.73 ± 0.86	1.82 ± 0.67	−1.70	0.090	−0.13
FAT (AU)	2.49 ± 0.86	2.40 ± 0.92	1.29	0.199	0.09
DOMS (AU)	2.40 ± 0.83	2.34 ± 0.90	1.04	0.297	0.07
HYD (AU)	2.75 ± 0.94	2.44 ± 0.76	4.92	<0.001	0.38
HI (AU)	11.47 ± 2.89	11.10 ± 2.73	1.83	0.068	0.13

Note. DOMS = delayed onset muscle soreness; FAT = fatigue; HI = Hooper Index; HYD = hydration status; RPE = perceived exertion; SLP = sleep quality; STR = stress. Data are presented as mean ± standard deviation (SD). Cohen’s *d* was used to estimate effect size. Statistical significance was set at *p* < 0.05.

## Data Availability

The data supporting the findings of this study are not publicly available due to privacy or ethical restrictions but can be provided by the corresponding author upon reasonable request.
